# Nutrient enrichment shapes depth-resolved bacterial community assembly in mangrove sediments

**DOI:** 10.1093/ismeco/ycag298

**Published:** 2026-10-28

**Authors:** Timothy Thomson, Marco Fusi, Natalie Prinz, S Craig Cary, Conrad A Pilditch, Joanne I Ellis

**Affiliations:** Environmental Research Institute, School of Science, University of Waikato, 101 Durham Street, Tauranga 3110, Bay of Plenty, New Zealand; Dove Marine Laboratory, School of Natural and Environmental Sciences, Newcastle University, Newcastle-upon-Tyne NE1 7RU, United Kingdom; Environmental Research Institute, School of Science, University of Waikato, 101 Durham Street, Tauranga 3110, Bay of Plenty, New Zealand; Environmental Research Institute, School of Science, University of Waikato, 101 Durham Street, Tauranga 3110, Bay of Plenty, New Zealand; Environmental Research Institute, School of Science, University of Waikato, 101 Durham Street, Tauranga 3110, Bay of Plenty, New Zealand; Institute of Marine Science, Faculty of Science, University of Auckland, Science Centre 302, Symonds Street, Auckland Central, Auckland 1010, New Zealand; Environmental Research Institute, School of Science, University of Waikato, 101 Durham Street, Tauranga 3110, Bay of Plenty, New Zealand

**Keywords:** mangrove sediment microbiome, community assembly processes, eutrophication, *Avicennia marina*, temperate mangroves

## Abstract

Microbial communities in mangrove sediments are critical for carbon and nutrient cycling; yet despite their importance, responses to eutrophication—a pervasive global threat to coastal ecosystems—remain poorly understood. Here, we tested whether nutrient enrichment restructures mangrove sediment bacterial communities more strongly in the subsurface than at the sediment surface. We assessed bacterial community responses to an in-situ slow-release NPK (Osmocote®) enrichment experiment in a temperate mangrove (*Avicennia marina* subsp. *australasica*) forest in Aotearoa New Zealand. Using 16S rRNA gene sequencing, we compared surface (0–2 cm) and subsurface (5–7 cm) community diversity and functional potential, 4.5 months after enrichment. Differences in porewater nutrient concentrations between ambient and enriched plots were subtle and spatially heterogeneous; however, bacterial communities exhibited significant shifts relative to controls. Community shifts in subsurface samples were stronger compared to the surface, with enrichment reducing diversity and altering community composition to *Proteobacteria* and *Bacteroidota* favoured communities. Conversely, taxa from *Desulfobacterota*, *Chloroflexi*, and *Nitrospirota* were more abundant in controls. Similarly, shifts in inferred functional potential in the surface remained limited, while the subsurface showed strong, selective changes: a proportional decline of sulphur-cycling and hydrogen-oxidizing taxa and a relative increase of heterotrophic guilds. Community assembly was more deterministic in subsurface compared to surface sediments, and under nutrient addition subsurface assembly shifted, mainly through reduced dispersal limitation, to more closely resemble the surface communities. These results underscore the need to better understand and integrate microbial responses at relevant spatial scales into predictions of ecosystem resilience under increasing anthropogenic pressures.

## Introduction

Mangrove forests are among the most productive ecosystems on the planet and are recognized for their capacity to sequester carbon, thereby mitigating climate change [1]. High productivity and slow decomposition under reducing and anoxic conditions drive the accumulation of carbon-rich organic matter in mangrove sediments [2]. Degradation processes that affect this accumulation are controlled by a diverse array of microbial metabolic pathways, highly adapted to the constraining and dynamic conditions present in intertidal sediments [3, 4].

Sediment microbiomes across mangrove forests globally are dominated by *Proteobacteria*, *Bacteroidota*, and *Desulfobacterota*. They are metabolically diverse and syntrophic, combining sulphate-reducing, fermentative, and chemolithoautotrophic taxa that couple carbon-, sulphur-, and nitrogen-cycling under fluctuating tidal, oxygen, and organic-matter conditions [5, 6]. Bacterial communities in mangrove sediments are shaped by tidal influence [7–9], nutrient and resource availability [10–13], pH [14], bioturbation [15], and tree species [16, 17]. Identifying how these communities respond to defined perturbations such as nutrient enrichment is central to anticipating the effects of anthropogenic pressure on organic-matter turnover and nutrient regeneration in mangrove sediments [18].

Coastal ecosystems are under enormous pressure from land-derived stressors due to ever increasing anthropogenic activities [19], which have been clearly linked to increased nutrient concentration in adjacent coastal sediments [20]. Particularly, the exposure of aquatic ecosystems to nutrient over-enrichment often leads to eutrophication with associated detrimental effects on species assemblages, ecosystem functions, and occasionally ecosystem collapse [21, 22]. Mangrove sediment microbial communities play a critical role in mediating the impacts of nutrient enrichment on ecosystem functioning [23, 24], yet how they specifically respond to enrichment remains poorly resolved [25, 26]. Because microbial communities turn over rapidly and respond to changing conditions well before larger ecosystem components, and often before critical thresholds are crossed, they can provide early and sensitive indicators of change [27, 28]. Soil microorganisms, for example, are expected to react swiftly to changes induced by nutrient input, with implications for gas exchange and overall carbon sequestration rates [29, 30]. Mechanistically, nutrient enrichment fosters the growth of opportunistic autotrophs, leading to the rapid production of labile organic carbon and subsequent proliferation of heterotrophic microorganisms and exoenzymes [30, 31]. More broadly, eutrophication has been shown to homogenize microbial communities, favouring generalist taxa and eliminating sensitive species [32–34]. Previous enrichment studies report reduced bacterial diversity and shifts in specific functional groups (e.g. decreased sulphate reducers, increased nitrogen cyclers) [24, 35–37], whereas others report diversity increases at impacted sites [38, 39]. The few controlled, in-situ enrichment studies that account for real-world complexity report small or environment dependent effects [37, 40, 41]. Furthermore, the high variability in these measurements and the dynamic nature of the intertidal mangrove habitat complicates the reproducibility of these results [42, 43].

One likely source of this variability is the strong vertical structuring of mangrove sediments. Steep physicochemical gradients across millimetres or centimetres create contrasting surface and subsurface environments that shape resident microbial communities as a function of depth [44–46]. Surface sediments are exposed to greater environmental variation, from fluctuations in light, oxygen, tidal water, and desiccation during emersion, favouring aerobic and phototrophic microbes. In contrast, subsurface sediments are typically anoxic, supporting anaerobic processes such as sulphate reduction and dissimilatory nitrate reduction [47, 48]. Despite this clear physical separation, depth-resolved nutrient enrichment effects on mangrove sediment microbial communities remain poorly resolved. Nutrient enrichment can restructure sediment bacterial communities, with total nitrogen and phosphorus driving significant reductions in alpha diversity [49] and dynamics of nitrogen cycling genes [50, 51]. Subsurface communities appear particularly responsive to phosphorus enrichment [52], consistent with theory that limiting nutrient conditions in deeper sediments impose strong selective pressures on microbial filtering [53]. Enrichment also shifts the balance between aerobic and anaerobic metabolic pathways, though the fate of sulphate-reducing and denitrifying bacteria remains context-dependent [48, 54], and enhances deterministic community assembly through increased homogeneous selection [55]. However, whether these effects on community assembly and functional potential vary with sediment depth remains untested.

This study investigates how experimental nutrient enrichment shifts microbial community composition, functional potential, and assembly processes in temperate mangrove sediments, and how this response differs between the surface and the subsurface. We expect nutrient enrichment to reduce bacterial diversity by favouring generalist taxa, with different groups affected in the surface (e.g. nitrogen cyclers, autotrophs) and the subsurface (fermenters, sulphur cyclers). Building on this, we test two mangrove-specific sub-hypotheses: (i) that the redox-constrained, less dynamic subsurface communities are more strongly and persistently restructured by enrichment than the environmentally variable surface communities; and (ii) that enrichment shifts the balance of community assembly processes, weakening the depth contrast in deterministic versus stochastic assembly that characterizes undisturbed mangrove sediments.

## Materials and methods

### Study site

The study site was located in the Whangamata estuary (37° 10.63′ S 175° 51.68′ E) on the east coast of the North Island of Aotearoa New Zealand (Fig. S1A). This barrier-enclosed estuary has a tidally driven hydrology over extensive areas of low-gradient intertidal flats. The estuary is subject to a semi-diurnal tidal cycle (mean spring-tide amplitude 1.8 m) and receives low freshwater inputs. Mean air temperature is 15.2°C with annual average rainfall of approximately 1360 mm. Mangrove stands of *Avicennia marina* subsp. *australasica* cover approximately 100 ha (~20% of the estuary) on the western shoreline.

### Experimental design, enrichment, and sampling

A fully randomized sampling design was established in the interior of the forest 15–20 m landward from the seaward fringe, positioned so that the tidal exposure time at each plot was similar (Fig. S1A). Plots (1 m^2^) were established under the canopy, with a minimum of 10 m between plots. Each treatment (control and enriched) was replicated 9 times at two depths (0–2 and 5–7 cm), resulting in 18 plots and 36 unique samples (Fig. S1B).

Our experiment used fertilizer to elevate mangrove sediment porewater nutrient concentrations to mimic correlations we observed in a previous study with human land use intensification in estuary catchments [20]. The fertilization of a small patch of mangrove sediment was used only as a model to infer microbial community responses to nutrient enrichment, and we do not suggest, or support, the fertilization of mangroves, which may have detrimental effects on this, or the surrounding habitats. Nutrient enrichment was performed in October 2020 with a slow-release fertilizer complex (Osmocote® Exact Standard 5–6 M; 15:9:12 N:P:K). The duration of nutrient release from the pellets was specified by the manufacturer to be between 5 and 6 months; this rating is calibrated for terrestrial soils, and release is likely faster in continuously waterlogged, tidally flushed sediments. An application rate of 200 g N m^−2^ (which corresponds to 120 g P m^−2^ and 160 g K m^−2^) was chosen, representing medium to high levels of enrichment concentrations found in eutrophic estuaries [56]. A series of 20 evenly spaced fertilizer doses of equal volume were applied to each plot. Briefly, a sediment core (Ø 3 cm) was removed to inject fertilizer pellets 10–15 cm into the sediment, after which the core was replaced to re-seal the hole [56]. Thus, the nutrients were introduced from below rather than from the overlying water column. The resulting upward diffusion gradient differs from a natural eutrophication event, where enrichment originates at the sediment–water interface (often including a large injection of labile organic carbon and sub-oxic conditions), but rather mimics the long-term build-up of nutrients in a chronically eutrophied system, where higher nutrient concentrations would be expected in the subsurface. Control plots were cored similarly without the addition of fertilizer, to control for the physical disturbance. Sampling was conducted in February 2021, 4.5 months after enrichment, to reflect the effects of a chronically elevated nutrient regime in mangrove sediments.

Samples for sediment parameters were collected using a 60 ml polyethylene syringe (Ø 3 cm). All cores were immediately separated into surface (0–2 cm) and subsurface (5–7 cm) partitions; the intermediate layer (2–5 cm) was deliberately omitted to maximize the contrast between the oxygenated surface and the reduced subsurface horizons, and we therefore make no claim to a continuous depth gradient. Three cores were taken from a random location in the plot, and the respective surface and subsurface partitions were each combined into one composite sample to account for small-scale local variability. Porewater samples were collected using Rhizon samplers (MacroRhizon, SoilMoisture Equipment Corp., CA, USA). Two porewater technical replicates were collected per plot and depth (subsequently averaged), whereas the 16S rRNA gene samples were collected as independent cores per plot (biological replicates) with PCR triplicates pooled at the amplification stage; the two sample types therefore differ in their replication structure. The fully randomized sampling design, combined with pooled sub-samples, controlled for spatial heterogeneity at the habitat and at the plot scale. Hence, control samples were assumed to represent the natural microbiome variability. All samples were kept in the dark and on ice and until sampling was completed (max. 3 h after collection). Salinity, pH, and redox potential were measured from the collected porewater using a handheld multimeter probe (ProSolo ODO/CT, YSI Inc. Yellow Springs, OH, USA). Sediment, porewater nutrient, and total alkalinity (TA) samples were frozen at −20°C while dissolved organic matter (DOC) samples were cooled at 4°C until analysis. Samples for 16S amplicon sequencing were collected as individual replicates using coring devices as described above. Each core was separated into surface and subsurface fractions and immediately homogenized on a clean tray with a sterile spatula before being sampled into sterilized tubes. Tubes were sealed with parafilm and stored on salt ice in the dark before being transferred to −80°C freezer immediately after the sampling was completed.

### Analytical methods

Sediment parameters (median grain size (GS), mud content, water content, sediment density, and organic matter fractions [i.e. total organic matter (TOM), labile OM, recalcitrant OM; via loss on ignition]) were analysed using standard methods (see Supplementary Information for a detailed description of each procedure). Dissolved nutrients, dissolved organic carbon (DOC), and total alkalinity (TA) were measured via flow-injection analysis, wet oxidation, and open-cell titration, respectively. Microbial biomass carbon (MB-C) was estimated via chloroform direct-extraction (see Supplementary Information for protocols and instrumentation).

### DNA extraction and library preparation

Total DNA was extracted from 0.21 [0.10–0.28] g of sediment (mean [range]), using the DNeasy® PowerSoil® Pro extraction kit (QIAGEN®, Hilden, Germany), and standardized to 2.5 ng/μL. The V4–V5 of the 16S rRNA gene was amplified using the universal earth microbiome project primers 515F/926R [57]. Reads were processed with DADA2 (*v1.28*) and taxonomy was assigned against SILVA (v138). Full details and parameters are provided in the Supplementary Information.

### Statistics

All analyses were conducted in the R statistical language *v4.3.1* [58] using RStudio [59], and figures were prepared using the ggplot2 package *v3.4.4* [60]. The environmental variables were tested for differences between depths and treatments using analysis of variance (ANOVA). Tukey’s honestly significant difference (TukeyHSD) test was applied to test for *P*-value adjusted pairwise comparisons if the interaction term was significant. ASV richness and genus level Shannon diversity were estimated from non-rarefied count data using breakaway *v4.8.*4 [61] and DivNet *v0.4.1* [62], respectively, which consider the compositional nature of the data. Beta diversity was calculated from the non-rarefied count data using Euclidean distance on the robust centred log-ratio (rclr) transformed data frame (robust Aitchison distance [63]) using base R and *vegan v2.6-4* [64] and was visualized as principal component analysis (PCA) plot. Permutational Analysis of Variance (PERMANOVA) was used to test the overall community composition across depth and treatment groups. A model-based approach (*mvabund v4.2.1* [65]) that correctly addresses the mean–variance relationship of count data was used to identify taxa that drove the observed shifts between depth and treatment communities using individual linear models. Differential abundance analysis was performed on the non-rarefied count data (agglomerated at genus level) using *the MaAsLin3 model* [66]. MaAsLin3 was fit as a single joint model across both depths, with Plot included as a random intercept to account for the split-plot design. Functional assignments were performed on the significant taxa using the FAPROTAX database *v1.2.*12 [67] via the microeco package *v1.16.0* [68]. Phylogenetic tree construction was performed with *ape v5.7-*1 [69], and phylogenetic diversity (PD), nearest taxon index (NTI), and net relatedness index (NRI) were calculated with the picante package *v1.8.2* [70]. The microbial abundance table was used to infer the mechanisms of bacterial community assembly by applying the phylogenetic bin-based null model methodology of the *iCAMP* package with the recommended settings [71].

## Results

### Physicochemical parameters of the sediment layers

The enrichment produced a mix of genuinely weak bulk-sediment responses alongside several clear, significant changes in porewater chemistry (Fig. 1, Fig. S2, Tables S1 and S2). Nitrate/nitrite, phosphate, and total alkalinity were all significantly higher under enrichment (*P* = .002, *P* < .001, and *P* = .019, respectively), and sediment labile organic carbon was also significantly elevated (*P* = .048), while TOM and microbial biomass carbon showed the same direction of change just short of our significance threshold (*P* = .073 and *P* = .080). We did not, however, see an increase in porewater DOC itself (*P* = .13). Ammonium concentrations were significantly lower at depth (*P* = .009) but showed no effect in response to the enrichment. Phosphate and total alkalinity both showed significant interactions between depth and treatment (*P* < .001, and *P* = .019, respectively), which were driven by the enriched treatment in the subsurface (Table S2).

**Figure 1:**
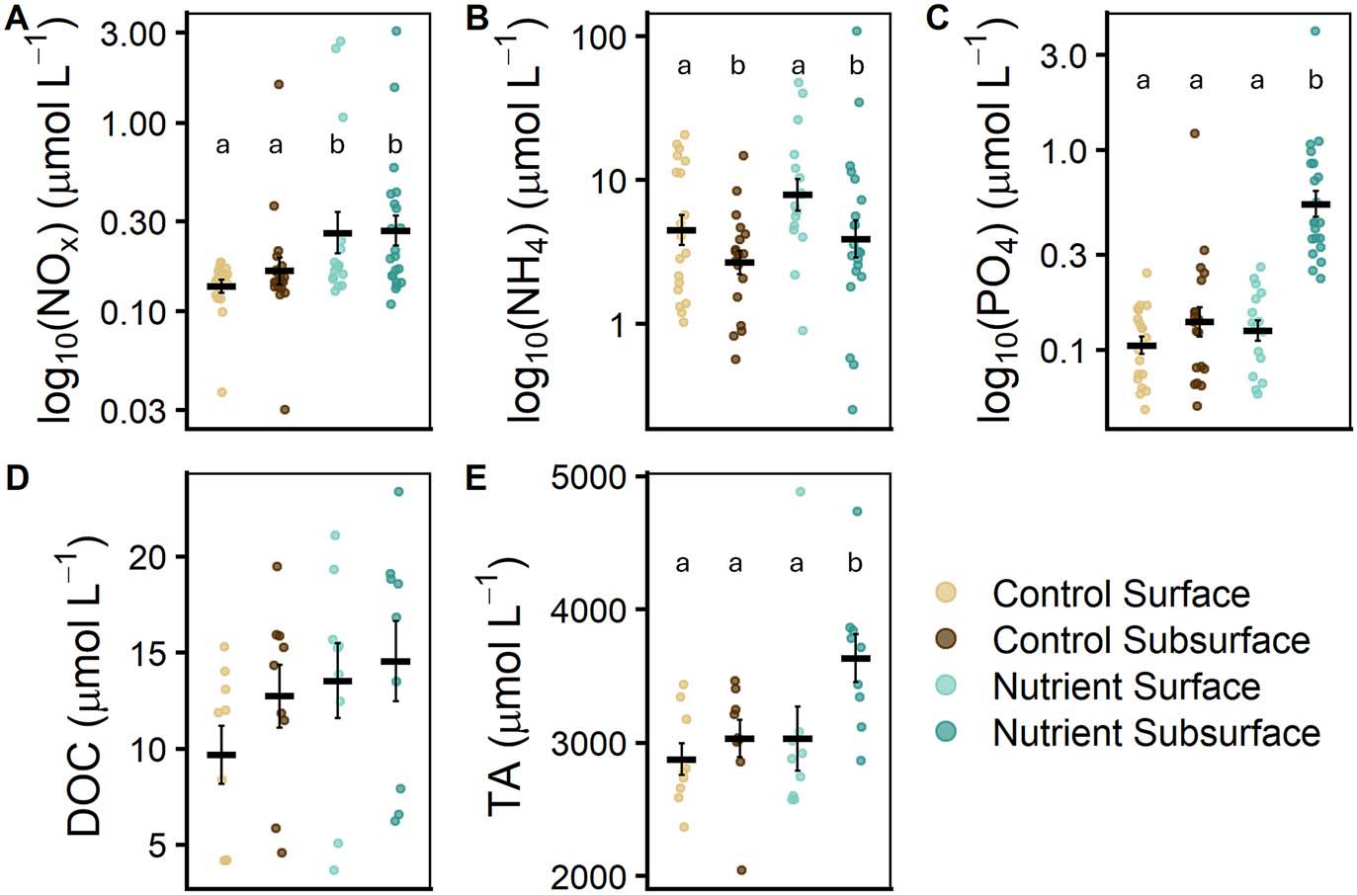
Porewater concentrations of (A) nitrate/nitrite (NO_x_), (B) ammonium, (C) phosphate, (D) dissolved organic carbon (DOC), (E) total alkalinity (TA), as a function of depth and treatment in mangrove sediments. The bars indicate the mean, and the error bars the standard error of each group (*n* = 18). Points display the underlying sample distribution. Lower case letters indicate statistical differences between levels. Where no such letters are displayed, no significances were detected.

### Sequencing output and taxonomic assignment

After quality read processing and filtering, 103 of the 108 sequenced samples were retained for analysis (control surface *n* = 26, control subsurface *n* = 24, nutrient surface *n* = 27, nutrient subsurface *n* = 26). The resulting non-rarefied table comprised 4820 ASVs spanning 34 phyla and 129 named genera. Taxonomic assignment was near-complete at the phylum level (99.9% of ASVs assigned) but considerably lower at the genus level (44.2% of ASVs assigned a named genus), and this resolution gap was near constant across all four treatment × depth groups (44.2%–45.7%), reflecting the inherent limits of short-amplicon 16S classification rather than a treatment- or depth-related artefact.

### Aotearoa New Zealand mangrove sediment microbiome

The bacterial communities of mangrove sediments in Aotearoa New Zealand were assessed in the control sediments. These differed between surface and subsurface samples and were dominated by the presence of a few abundant and a long tail of rare taxa (Fig. S3). In control samples, approximately one third of ASVs were unique to each depth, with the remainder shared between depths (4036 ASVs total, 32 phyla, 118 genera). Subsurface communities had substantially higher ASV richness than surface communities (+99.4 taxa, *P*-value <.001; Fig. 2A, Table S3), while being less diverse (Shannon Index) when aggregated at genus level (−0.17, *P*-value <.001; Fig. 2A, Table S3). This difference between depth layers in the control samples was visualized by robust Aitchison principal component analysis (RPCA; Fig. 2B), and multivariate analysis of the bacterial community composition confirmed a significant difference at depth (mvabund; likelihood ratio test (LRT) = 8590.4, *P*-value <.001, Table S4). At the sediment surface, most ASVs belonged to the phylum of *Proteobacteria* (relative abundance, 36.5%), followed by *Bacteroidota* (24.1%), and *Desulfobacterota* (22.1%). All other phyla made up the remaining 17.3% with no single contribution above 5% (Fig. 2C). At class level, *Gammaproteobacteria* (31.4%), *Bacteroidia* (23.2%), and *Desulfobacteria* (11.1%) were dominant across samples. The same phyla and classes dominated the communities in the subsurface; however, the dominance order changed to *Desulfobacterota* (33.8%; *Desulfobacteria* 23.6%), *Proteobacteria* (24.3%; *Gammaproteobacteria* 21.9%), and *Bacteroidota* (18.6%; *Bacteroidia* 17.8%). The remaining 23.3% of phyla in the subsurface were led by *Chloroflexi* (6.9%) and *Acidobacteriota* (4.1%) (Fig. 2C).

**Figure 2:**
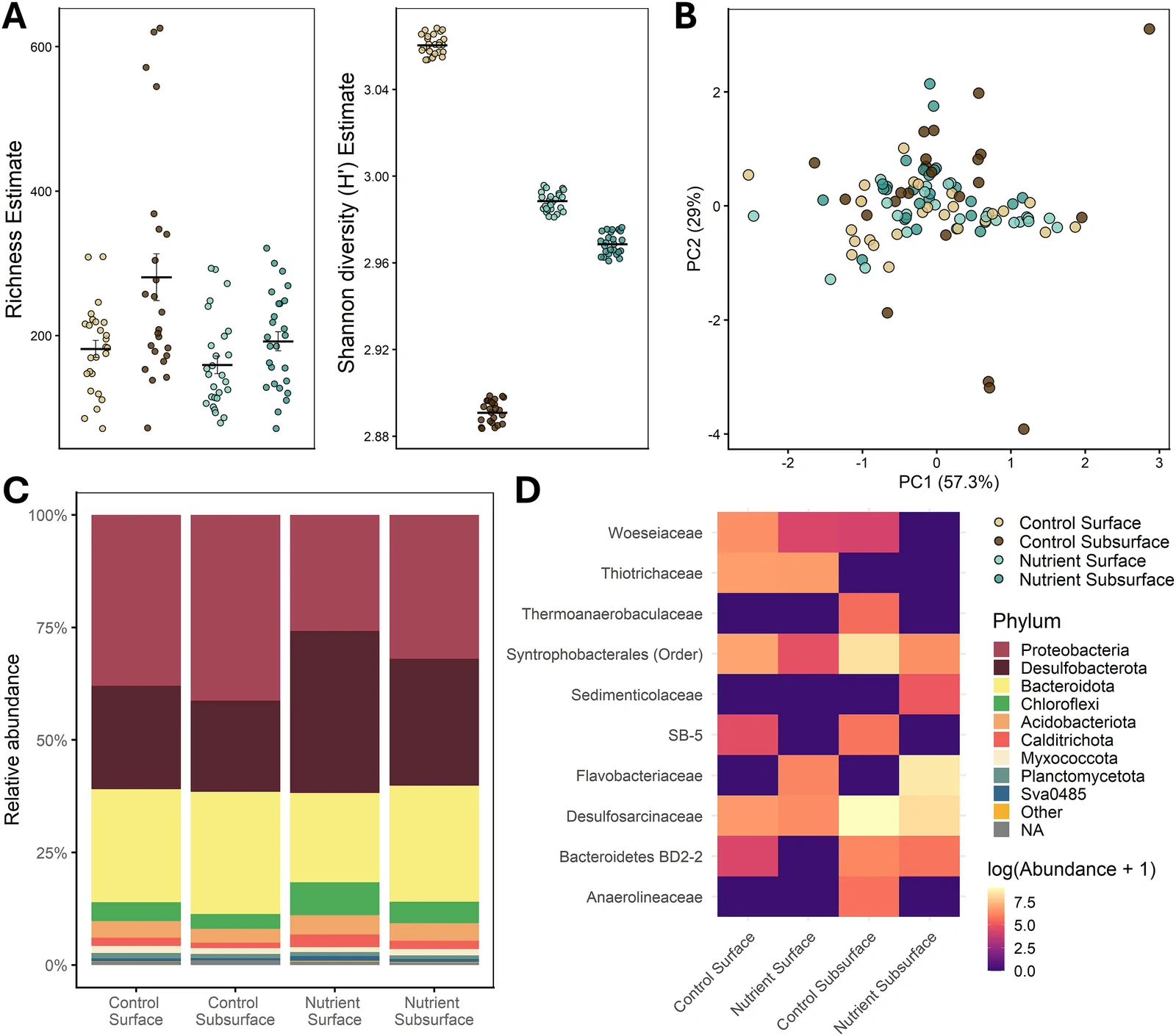
Characterization of differences in the bacterial communities between mangrove surface and subsurface sediment samples from control and nutrient enriched plots. (A) Alpha diversity, expressed by the modelled number of observed ASVs (via breakaway) and the estimated Shannon diversity index on genera (via DivNet). The horizontal bars indicate the mean, and the error bars the standard error of each group (*n* = 27). Points show the underlying sample distribution. (B) RPCA plot showing the differentiation of the two communities (beta diversity). (C) Relative abundance of taxa at phylum level showing the top 10 most abundant phyla (remaining grouped as “Others”). (D) Taxa at family level, that drive the differentiation between treatments in the surface and subsurface layers as identified by the univariate adjustment of the mvabund algorithm.

### Effects of nutrient enrichment on the microbiome

The proportion of ASVs shared between surface and subsurface sediments from the enriched samples was similar to that of the control samples. Out of 3890 ASVs (34 phyla, 119 genera), approximately one-third (~32.5%, 1263 ASVs) were unique to the surface and another third (~32.5%, 1265 ASVs) unique to the subsurface layers, while the remaining ASVs (~35.0%, 1362) were shared between the two depths. Alpha diversity varied significantly by depth and treatment. Nutrient addition alone had no significant effect on richness at the surface (−22.2 taxa, *P*-value = .119), but the significant interaction (Depth × Treatment; −66.7, *P*-value = 0.001) indicates a much stronger negative nutrient effect in subsurface samples. Nutrient addition reduced Shannon diversity at the surface and increased it in the subsurface (relative to subsurface control), indicating an approximation of the communities under nutrient enrichment (Depth × Treatment; +0.150, *P*-value <.001; Fig. 2A; Table S3). Robust Aitchison-distance-based principal component analysis (RPCA) revealed that microbial community composition was significantly structured by the interaction between depth and nutrient treatment (PERMANOVA; Depth × Treatment: *R*^2^ = 0.030, *F*_1,99_ = 3.20, *P* = .038), indicating that the compositional response to nutrient addition depended on sediment depth. This interaction explained a modest proportion of total variance (3%; Table S5), consistent with the considerable overlap among all four groups in ordination space (Fig. 2B). Multivariate dispersion did not differ between depth groups (betadisper; *F*_1,101_ = 0.20, *P* = .658). Dispersion did differ significantly between treatments (*F*_1,101_ = 5.09, *P* = .026), driven largely by a small number of high-dispersion outliers within the control-subsurface samples (Fig. 2B), suggesting that the observed treatment effect may be affected by unequal sample spread. The model-based multivariate test (mvabund) confirmed the significant interaction effect between nutrient enrichment and sediment depth (LRT = 4803, *P*-value <.001; Table S4). However, the bacterial community composition varied much less between treatments in the surface layers (pairwise test; LRT = 5066, *P*.adj. = .032) than in the subsurface layers (pairwise test; LRT = 6571, *P*.adj.= .002). Depth within each treatment also appeared as an important predictor of community composition with the differences in control samples (LRT = 7192, *P*.adj. = .001) being more pronounced than in the enriched samples (LRT = 5856, *P*.adj. = .002).

Based on the univariate model results per taxa (mvabund; Fig. 2D), nutrient additions were associated with a higher relative abundance of Flavobacteriaceae, though to a lesser extent, while several common surface taxa, including SB-5, Bacteroidetes BD2-2, and Syntrophobacterales, declined in relative abundance with nutrient addition. *Thiotrichaceae* and *Woeseiaceae* remained comparatively stable at the surface regardless of treatment. In the subsurface, the families identified as statistically different between treatments (by mvabund) were generally more abundant in the control samples except for *Flavobacteriaceae* and *Sedimenticolaceae*, which were more abundant in the nutrient treatments (Fig. 2D). Differential abundance (MaAsLin3) confirmed the stronger subsurface response: out of 131 taxa (at genus level; aggregated from ASVs within MaAsLin3) retained after internal prevalence filtering, the model identified zero taxa with a significant treatment effect at the surface, and eight taxa with a significant treatment effect at the subsurface (Fig. 3; Table S6). An additional four taxa showed a significant depth × treatment interaction effect, indicating that the treatment response of these four taxa differed significantly between surface and subsurface sediments (Fig. 3). Depth alone identified 24 taxa as significantly different between surface and subsurface sediments.

**Figure 3:**
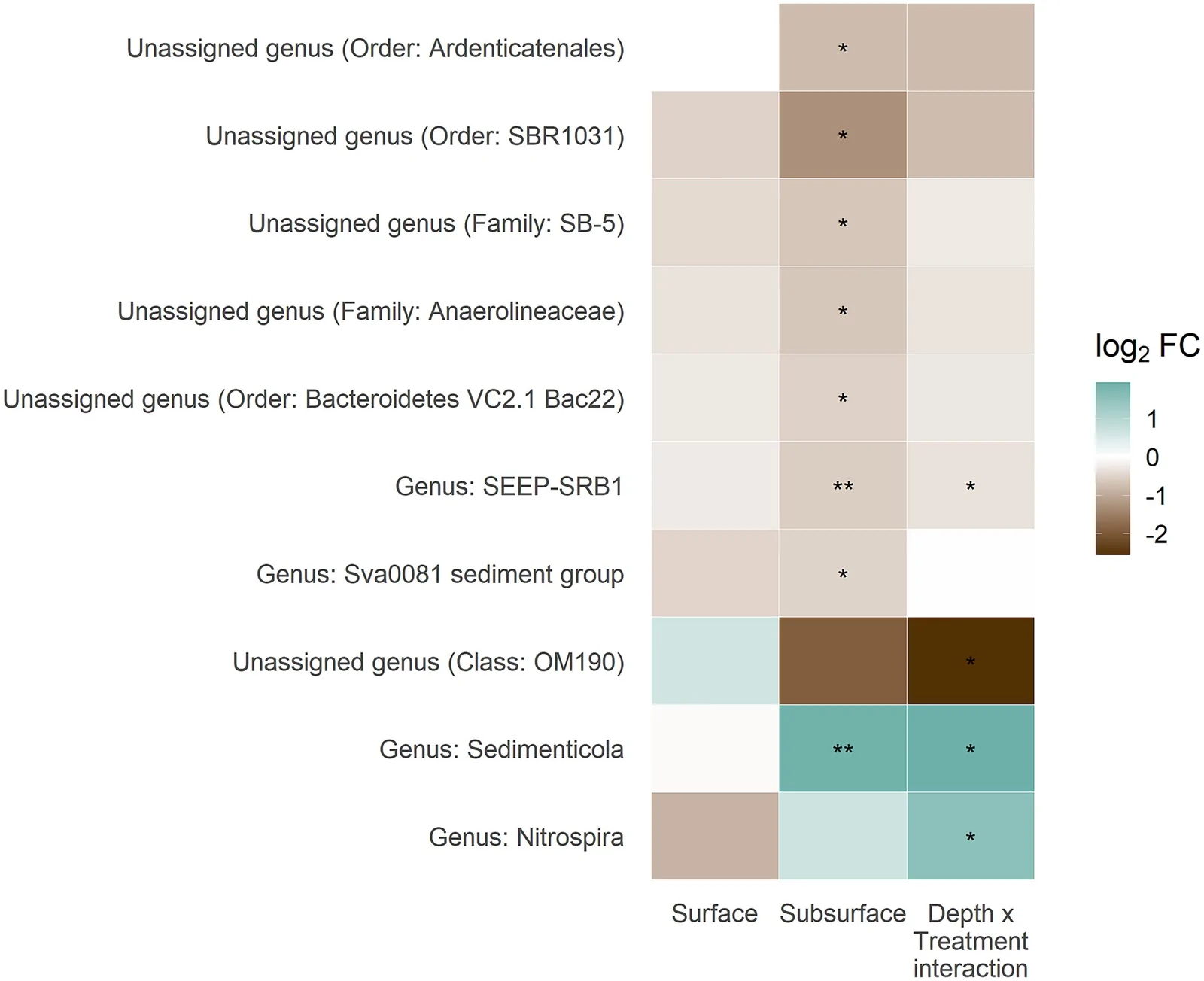
Heatmap showing the differentially abundant taxa (at genus level) identified by MaAsLin3. Nearest taxonomic rank is shown where the taxonomy was not resolved at genus level. the first two columns show the treatment effect at each sediment depth, while the last column shows the interaction effect between depth and treatment. The colour indicates the direction (teal = more abundant in enriched plots; brown = more abundant in control plots) and the strength (log_2_-fold change) of the effect. Asterisks indicate the significance of each effect (Benjamini–Hochberg corrected *q*-value: ^*^ < 0.1, ^**^ < 0.01).

The functional assignments (FAPROTAX) on the significantly different taxa revealed sulphate respiration (and respiration of sulphur compounds), dark hydrogen oxidation, anaerobic chemoheterotrophy, and chemoheterotrophy to be substantially greater in control subsurface sediments (Table 1).

**Table 1:** Functional assignments (FAPROTAX) of significantly different taxa (at genus level) between nutrient enriched and control samples in surface and subsurface sediment; nearest taxonomic rank is shown where the taxonomy was not resolved at genus level.

**Genus**	**Significant effect**	**FAPROTAX function(s)**
**Unassigned genus (Order:* Ardenticatenales*)**	Higher in control subsurface treatment (Subsurface)	—
**Unassigned genus (Order:* SBR1031*)**	Higher in control subsurface	—
**Unassigned genus (Family:* SB-5*)**	Higher in control subsurface	—
**Unassigned genus (Family:* Anaerolineaceae*)**	Higher in control subsurface	Anaerobic chemoheterotrophy; chemoheterotrophy
**Unassigned genus (Order:* Bacteroidetes VC2.1 Bac22*)**	Higher in control subsurface	—
*SEEP-SRB1*	Higher in control subsurface; depth × treatment interaction	Anaerobic chemoheterotrophy; chemoheterotrophy; dark hydrogen oxidation; respiration of sulphur compounds; sulphate respiration
*Sva0081 sediment group*	Higher in control subsurface	Anaerobic chemoheterotrophy; chemoheterotrophy; dark hydrogen oxidation; respiration of sulphur compounds; sulphate respiration
**Unassigned genus (Class:* OM190*)**	Depth × treatment interaction	—
*Sedimenticola*	Higher in nutrient subsurface; depth × treatment interaction	—
*Nitrospira*	Depth × treatment interaction	—

### Assembly mechanisms highlight differences between depth layers

Differences in phylogenetic patterns between samples were assessed using the NTI and NRI scores [72]. The NTI scores <1.96 indicated a dominance of stochastic processes across all samples with only marginally higher values in the subsurface samples compared to the surface samples (*F*-test_1,99_ = 6.64; *P*-value = .01, Fig. 4A; Table S7). The factor treatment showed no significant effect (*F*-test_1,99_ = 1.39; *P*-value = .24). The NRI score showed a significant interaction effect between treatment and depth (*F*-test_1,99_ = 4.58; *P*-value = .03, Fig. 4B; Table S7) which was driven by differences between depths of the control samples (TukeyHSD; *P*.adj. < .001) and differences between treatments in the subsurface layer (TukeyHSD; *P*.adj. < 0.001).

**Figure 4:**
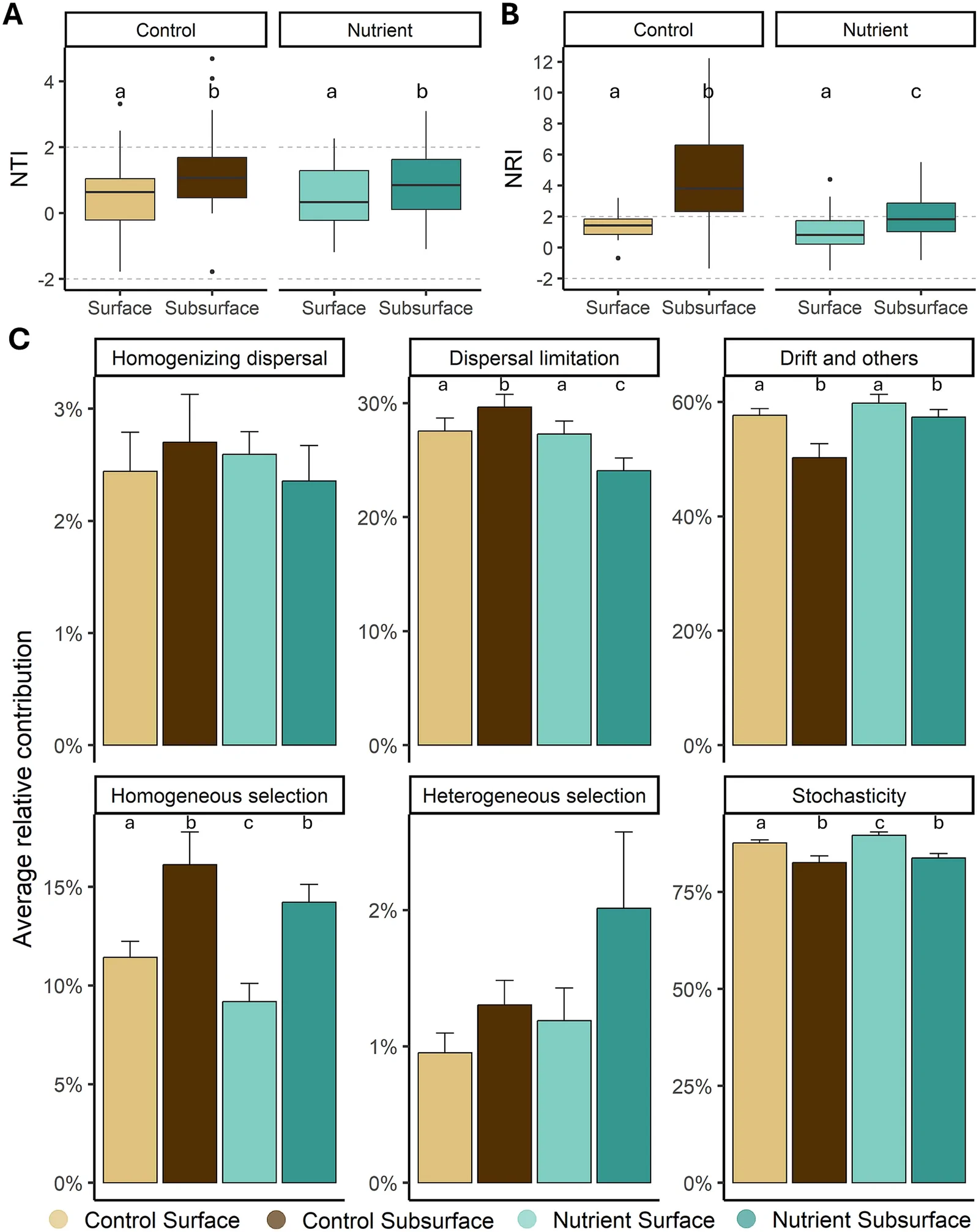
Patterns of phylogenetic diversity structure of the bacterial community from different depths and treatments. (A) Nearest taxon index (NTI) is calculated from the mean distance to the nearest neighbour on the phylogenetic tree. (B) Net relatedness index (NRI) computes the mean pairwise distances between each taxon on the phylogenetic tree. Both indexes are fitted against null models to give a standardized measure of effect size of phylogenetic clustering or overdispersion. The area between the two dashed lines indicates that stochastic processes dominate. The boxplots indicate the median with the interquartile range (IQR) between the 25th and the 75th percentile and the whiskers extend 1.5^*^IQR. (C) Average contributions of bacterial community assembly processes. These processes were calculated from distance-based phylogenetic structure analysis following the iCamp methodology. Stochastic assembly processes (top row) are summed to calculate stochasticity (bottom right). Homogeneous and heterogeneous selection are deterministic processes. Bars and errors represent means and standard deviations of the permutational analysis.

The stochastic processes of drift and dispersal limitation were the most dominant assembly mechanisms identified by the iCAMP methodology with relative contributions ranging between 41.7%–64.5% and 23.7%–32.9%, respectively, across all treatments and layers (Fig. 4C). Of the deterministic mechanisms, homogeneous selection was more dominant than heterogeneous selection across all treatments and layers (relative contribution, 6.7%–22.9% and 0.5%–4.4%, respectively). The surface samples differed significantly from the subsurface samples (Fig. 4C; Table S8). In the surface samples, effects of the nutrient treatment were only detectable in the homogeneous selection, which showed a lower relative contribution in the nutrient samples (Cohen’s *d* = 2.6, *P*-value = .03, Table S8). In the nutrient treatment, communities showed a significantly higher ratio of drift (Cohen’s *d* = −3.6; *P*-value = .005), but a lower ratio of dispersal limitation (Cohen’s *d* = 5.0; *P*-value <.001). The stochasticity (all stochastic processes combined: homogenizing dispersal, dispersal limitation, and drift and others) explains between 75.8% and 92.0% of community assembly mechanisms from all samples. The stochastic processes are more important in the surface communities than in the subsurface, which is consistent across the two treatments (control, Cohen’s *d* = −3.8; *P*-value = .001; nutrient, Cohen’s *d* = −6.0; *P*-value <.001; Table S8). However, the effect of the nutrient treatment in the subsurface is masked when assessing the combined stochasticity (Cohen’s *d* = −0.8, *P*-value = .3).

## Discussion

In this study, we assessed the sediment bacterial community response to a manipulative in-situ nutrient enrichment experiment in a temperate *A. marina* subsp. *australasica* mangrove forest in Aotearoa New Zealand. This study represents the first characterization of the bacterial community of Aotearoa New Zealand mangroves, and to our knowledge only the second (after [37]) to characterize the bacterial community composition in temperate mangrove sediments worldwide. Our results indicate a distinction between community responses to nutrient enrichment inhabiting the surface (0–2 cm) and the subsurface (5–7 cm) layers. We observed community-level differences due to nutrient enrichment at both depths with a stronger effect in the subsurface layer, indicating prolonged disturbance effects in this less dynamic environment, suggesting an increased sensitivity, or responsiveness, of these sheltered bacterial communities to nutrient enrichment.

Mangrove prokaryote communities vary substantially by bioregion [6]. Despite this variation, the control (unenriched) communities in this study were dominated by phyla consistently reported globally as characteristic of mangrove sediment (Phyla: *Proteobacteria, Bacteroidota,* and *Desulfobacterota*) [5, 6], indicating that temperate Aotearoa New Zealand mangrove sediments broadly resemble global compositional patterns at the phylum level. The subsurface community contained more taxa overall (higher richness) but was less even, with lower Shannon diversity than the surface. This indicates that while more taxa accumulate at depth, the subsurface community is dominated by a smaller number of abundant taxa. This pattern is consistent with the more stable conditions typical of deeper mangrove sediment, where reduced physical mixing allows well-adapted specialists to dominate, most notably sulphate-reducing *Desulfobacterota*, which dominated our subsurface samples (33.8% relative abundance). Furthermore, strong environmental filtering through spatially heterogeneous resource distribution may also contribute to this pattern [73]. The surface community, by contrast, is subject to greater tidal mixing and exchange with the overlying water column, which reduces the dominance of any single group, making the community more even.

The subsurface dominance of *Desulfobacterota* we observe is also consistent with recent evidence that deeper mangrove sediment layers host chemolithoautotrophic taxa, including *Desulfobacterota*- and *Halobacteriota*-affiliated diazotrophs, that are functionally distinct from the shallower nitrogen-fixing assemblage dominated by *Campylobacteria* and *Gammaproteobacteria* [74], which means that the community differentiation by depth likely corresponds with a functional shift in microbial metabolisms. However, whether the depth-related compositional shifts translate into different rates of organic matter turnover, nitrogen fixation, or sulphur cycling would require complementary metatranscriptomic or flux-based approaches, which present a priority for future work in this system.

### Nutrient enrichment more strongly affects the subsurface community

The level of enrichment, assessed by porewater concentrations of dissolved nutrients, was not as strong as expected based on previous studies using the same methodology [56, 75]. This may be due to the longer incubation period chosen for this study (4 months versus 10 weeks) or differences in system properties such as sediment grain size and inter-sedimentary hydrodynamic flow. While the period for continuous nutrient enrichment was specified as 5–6 months (by the manufacturer), this may be greatly reduced for use in waterlogged, dynamic coastal sediments, consistent with field studies showing that controlled-release fertilizer N-release rates in permanently flooded paddy soils exceed both laboratory and manufacturer-rated release periods, likely because soil moisture is never limiting under flooded conditions [76]. Alternatively, the enrichment method used may have induced high local variability in nutrient concentrations, which was not captured by the porewater sampling design. This would also explain the very high outliers measured in the porewater of some nutrient plots. We therefore interpret the enrichment as partial and spatially heterogeneous and treat the community-level responses below as conservative estimates of the effect of nutrient addition.

Despite the heterogeneous signal of an enrichment effect, the microbial community differed significantly between treatments. This difference was especially pronounced in the subsurface samples, while the surface samples showed only weak, or no differences between the treatments. This depth-dependent divergence is consistent with the niche partitioning typical of coastal sediments, which structures microbial communities and selects for highly specialized and adapted organisms in the subsurface [45]. Our results confirm this spatial compartmentalization along a vertical depth profile and highlight the defining influence this has on the microbial communities inhabiting each depth layer, likely due to hydrodynamic forcing and advective porewater flow [77]. In dynamic shallow coastal environments, hydrodynamic disturbance (tides, waves) tends to mix the upper layers and increase exchange of solutes and organisms with the water column, homogenizing sedimentary microbial communities in the boundary layer [9, 78–80], whereas deeper layers are more redox-constrained, favouring specialized anaerobic metabolisms [51]. We note, however, that measured ORP ranged only between approximately −29 and + 42 mV with no clear vertical stratification, indicating generally suboxic conditions throughout the sampled profile. The community-level depth contrast is therefore better attributed to cumulative niche partitioning and reduced physical mixing at depth than to a sharp redox boundary. Because the nutrients were delivered at 10–15 cm, closer to the subsurface horizon, the stronger subsurface response may reflect proximity to the nutrient source in addition to genuine depth-related sensitivity.

The depth-dependent effects of nutrient enrichment on alpha diversity indicate different underlying mechanisms at each depth. At the surface, ASV richness remained similar under enrichment, while genus-level Shannon diversity declined, indicating that a small number of genera increased disproportionately reducing evenness. In the subsurface, ASV richness declined under nutrient enrichment, while genus-level Shannon diversity increased relative to the control, bringing the subsurface nutrient community’s genus-level diversity close to that of the enriched surface community. This increase in genus-level diversity, despite falling ASV richness, suggests that enrichment relaxes environmental filtering in the subsurface. It is important to note, that ASV richness and Shannon diversity were assessed at different taxonomic resolutions (with only 44.2%–45.7% of ASVs resolved to genus rank), which means that the two should not be interpreted as reflecting the same underlying community pattern. Nutrient enrichment has been reported to decrease microbial diversity in a range of ecosystems, through the alleviation of selective pressure, giving a competitive advantage to a few generalist taxa [81, 82]. Our results suggest this dynamic depends on the nature of the environmental conditions: concentrating dominance within the more stochastically assembled surface community, while loosening the selective constraints that structure the specialist-dominated subsurface community.

Differential abundance analysis identified no genera significantly associated with nutrient treatment at the surface, which is consistent with the alpha and beta diversity results. The observed changes at depth, showed taxa from the *Chloroflexi* (*Anaerolineaceae*, *Ardenticatenales*, *SBR1031*) and *Desulfobacterota* (*SEEP*-*SRB1*, *Sva0081* sediment group) to be more abundant in control samples. These groups are characteristically slow-growing anaerobes reliant on narrow redox and electron-acceptor niches [83–85]. Their suppression under enrichment is consistent with competitive displacement by faster-growing, copiotrophic taxa able to exploit the additional resource supply more rapidly. However, *Sedimenticola* (*Gammaproteobacteria*), a sulphur-oxidizing genus, became markedly more abundant under nutrient addition at depth. This divergence suggests nutrient enrichment reshapes which part of the sulphur cycle is favoured, rather than dampening the entire sulphur cycle.

Functional annotation of significant taxa identified by differential abundance analysis revealed clear depth-dependent patterns in how nutrient enrichment altered functional community profiles. Because FAPROTAX annotates only a subset of the detected taxa and infers functional potential rather than realized function, these assignments provide coarse, indicative functional patterns rather than confirmed function. Functional coverage was also limited by taxonomic resolution: several of the differentially abundant genera could not be resolved beyond Family or Order level and were therefore excluded from FAPROTAX matching, since function assignment requires genus- (or species-) level identity. Furthermore, at least one fully resolved, well-characterized genus (Nitrospira, a documented nitrite oxidizer, which showed a significant depth × treatment interaction) likewise returned no assignment, due to a naming mismatch between the FAPROTAX reference database (v1.2.12, which retains the pre-2021 phylum name Nitrospirae) and the SILVA *v138* taxonomy used here (Nitrospirota). Taxonomic shifts in the surface layer did not result in significant shifts in inferred functional potential, consistent with (though not direct evidence of) functional redundancy in surface sediments, potentially due to a more generalist community and/or rapid exchange with overlying tidal water [86]. In contrast, the changes in the subsurface communities led to significant alterations of their functional potential. Sulphur-cycling and hydrogen-oxidizing taxa (together with anaerobic chemoheterotrophs) were disproportionally suppressed in enriched samples, suggesting that nutrient addition can alter carbon and hydrogen metabolisms at depth. Our results suggest that natural, nutrient-poor sediments support more diverse and functionally complex microbial networks. Nutrient inputs reduce community diversity by favouring fast-growing opportunists at the expense of slower, specialized anaerobes. This depth-stratified sensitivity has important implications for sediment biogeochemistry under eutrophication, including altered carbon degradation pathways and reduced sulphur-cycling capacity. Given the suboxic conditions and elevated NO_x_ in enriched plots, the proportional loss of sulphate-reducing taxa may also favour nitrogen-based respiratory pathways such as denitrification and dissimilatory nitrate reduction to ammonium (DNRA) over sulphate respiration [50], although this inference rests on taxonomic (DNA) data and would require direct rate or transcript measurements to confirm.

Nutrient enrichment in mangrove sediments can destabilize blue carbon through introduction of alternative electron acceptors (e.g. nitrate), stimulation of microbial biomass and enzyme production, and disruption of physical and chemical protection mechanisms [87]. Our observations of reduced sulphur-cycling taxa and altered functional potential in nutrient-enriched subsurface sediments theoretically support this destabilization mechanism, suggesting that nutrient-driven changes in microbial metabolism could compromise long-term carbon storage.

### More deterministic bacterial community assembly processes in the subsurface layers

Microbial communities are shaped by assembly processes that are governed by ecological interactions of stochastic and deterministic nature [88]. Selective pressure (homogeneous, heterogeneous) is applied through environmental forcing, while stochastic processes relate to randomly acting factors such as dispersal limitation and genetic drift [73, 88]. Recognizing which processes dominate in mangrove sediments and how they may change in response to shifting environmental conditions is important for understanding and predicting microbial community change resulting from anthropogenic stressors [73]. The dominance of stochastic community assembly mechanisms observed here, and in particular phylogenetic drift, in mangrove sediments corroborates findings of previous studies [8, 89]. While stochastic processes remain dominant across all samples, they decrease significantly in the subsurface compared to the surface samples, which is likely due to the increased specificity of the subsurface community regarding niche space and resource limitation [45, 90]. The tidally mixed surface layer is likely to receive frequent inputs from porewater, overlying water, and adjacent sediment patches, while also experiencing short-term fluctuations in oxygen, salinity, and resource availability. Such conditions favour the coexistence (and replenishment) of multiple taxa capable of similar metabolisms rather than the competitive exclusion of all but one specialist [73, 89]. This functional redundancy should weaken deterministic selection and allow stochastic processes such as ecological drift and dispersal-driven replacement to contribute more strongly to surface community assembly, consistent with reports that abundant, rare, and generalist mangrove sediment taxa are predominantly stochastically assembled [91]. In contrast, the less physically mixed subsurface has fewer opportunities for replenishment and is structured by more persistent resource and redox constraints. Here, functional roles may be concentrated in fewer specialized taxa, such as sulphate-reducing *Desulfobacterota* [91]. Loss or proportional decline of these groups under enrichment would therefore be expected to have a stronger effect on community structure and to shift assembly towards deterministic filtering by the new nutrient regime [8, 90].

Studies from various terrestrial systems report a much lower influence of stochasticity (range 20%–55%) than rates reported from mangrove forests (range: 55%–92%) [92–95]. Interestingly, other studies from aquatic systems (lake and subtidal coastal sediments) also report relatively low contributions of stochasticity to bacterial assembly processes (20% and 30%–60%, respectively), which suggests that high stochasticity may be a tidally influenced phenomenon [96, 97]. West and Whitman [98] were able to demonstrate that even infrequent mixing of soil samples resulted in a substantial increase in stochastic assembly processes in microbial communities. This likely explains the observed differences between the “conserved” subsurface and the “disturbed” surface layers in mangrove forests, as well as the homogenizing influence of tidally driven water movement in intertidal areas. The depth-related differences in assembly mechanisms were weakened (or reversed) in the nutrient treatments, leading to assembly patterns in the subsurface of the nutrient samples that were more similar to those of the surface samples. This is most directly interpreted as a change in dispersal limitation rather than evidence of overall homogenization through homogenizing dispersal or selection; nonetheless, it is consistent with the idea that an excess of a limiting resource can relax the niche constraints that normally differentiate the subsurface community [99]. Decomposing these effects by individual process shows that nutrient addition significantly reduced homogeneous selection at the surface, raising the relative contribution of stochastic processes, despite unchanged physical mixing. This may be due to the patchy, localized nature of the enrichment signal reported above, which may have introduced heterogeneity at the surface level, despite the homogenizing force of tidal mixing. At depth, nutrient addition significantly reduced dispersal limitation while significantly increasing drift, with no corresponding change in homogeneous selection, indicating that enrichment altered which stochastic process dominates community reassembly rather than relaxing the deterministic environmental filter. These two opposing shifts largely offset one another in the combined stochasticity metric, which is why no net treatment effect was detected when the stochastic processes were considered together. As our design captures a single time point within one estuary, these patterns most directly reflect a local shift in assembly balance, which future multi-site or multi-timepoint studies could test for generality.

## Conclusion

In this study, we show that the contrasting physicochemical environments of mangrove surface and subsurface sediments resulted in varying responses of the sediment microbiome to nutrient enrichment. While the community of the dynamic, tidally disturbed surface sediments showed little response to nutrient additions after 4.5 months, the subsurface community composition shifted significantly between treatments, highlighting the vulnerability of less dynamic environments to disturbance. The altered sulphur- (and associated carbon-cycling) potential we observe at depth, together with the elevated total alkalinity and labile organic carbon in the enriched subsurface porewater and sediment [100], point towards cryptic consequences of nutrient enrichment for subsurface biogeochemical cycling in mangrove sediments, with potential relevance to other slowly mixed, disturbance-sensitive systems such as deep-sea sediments and cold-region soils. These functional links remain hypothetical, based on taxonomic (DNA) data, rather than direct rate or transcript measurements, and should be tested with targeted *in-situ* and laboratory studies. Our experiment used nutrient enrichment only as a model to infer microbial responses to anthropogenic nutrient loading; the deliberate application of fertilizers to mangroves would be ecologically detrimental and could undermine blue-carbon storage and adjacent ecosystems. More broadly, this study provides a first characterization of the bacterial community composition of temperate Aotearoa New Zealand mangrove sediments, extending the global understanding of these ecologically important habitats. Furthermore, it underscores the value of depth-resolved, spatially explicit monitoring for predicting how mangrove microbial communities, and the biogeochemical functions they support, will respond to increasing anthropogenic pressure.

## Supplementary Material

Supplementary_Materials_ycag298

## Data Availability

The raw sequence data and associated metadata were deposited in the SRA of the NCBI (https://www.ncbi.nlm.nih.gov/sra) under accession number PRJNA1433135.

## Dedication

I dedicate this work to my mentor Stephen Craig Cary, who sadly passed away a few months before the completion of my PhD. Craig has been an enormously positive influence on me in the three years that we have worked together. He was an inspiring presence, and his enthusiasm for science and scientific discovery was contagious. Craig would always find some time in his busy schedule to talk about my work, and he encouraged me to think big and to think independently. His trust in me as a researcher was something that carried me through some of the harder parts of this PhD journey, and I am grateful to him for that.
