# Supplementary material for: Nutrient enrichment shapes depth-resolved bacterial community assembly in mangrove sediments
Source: ISME Commun. 2026 Oct 28;6(1):ycag298. doi: 10.1093/ismeco/ycag298 (PMC13747022; doi:10.1093/ismeco/ycag298)
Supplement: Supplementary file 1 [file supplementary_materials_ycag298.zip › Supplementary_Materials_ycag298/Supplementary_file_final_ycag298.docx]

**Nutrient enrichment shapes depth-resolved bacterial community assembly in mangrove sediments**

**Running head: Nutrient Effects in Mangrove Sediments**

Timothy Thomson^a*^, Marco Fusi^b^, Natalie Prinz^a^, S. Craig Cary^a^, Conrad A. Pilditch^a,c^, Joanne I. Ellis^a^

^a^Environmental Research Institute, School of Science, University of Waikato, 101 Durham Street, Tauranga 3110, Bay of Plenty, New Zealand

^b^Dove Marine Laboratory, School of Natural and Environmental Sciences, Newcastle University, Newcastle-upon-Tyne NE1 7RU, United Kingdom

^c^Institute of Marine Science, Faculty of Science, University of Auckland, Science Centre 302, Symonds Street, Auckland Central, Auckland 1010, New Zealand

*corresponding author: Timothy Thomson, Environmental Research Institute, School of Science, University of Waikato, 101 Durham Street, Tauranga 3110, Bay of Plenty, New Zealand, timothy.thomson@waikato.ac.nz

**Supplementary methods**

**Sampling**

Sampling was conducted in February 2021. Samples for sediment parameters were collected using a 60 mL polyethylene syringe (Ø 3 cm) with cut-off tip that extended approximately 10 cm into the sediment. All cores were immediately separated into surface (0 - 2 cm) and subsurface (5 – 7 cm) partitions. Three cores were taken from a random location in the plot, and the respective surface and subsurface partitions were combined into one composite sample to account for small-scale local variability. The composite sample was subsequently separated into sampling containers for distinct measurements (1) Total organic matter (TOM) & density, 2) sediment grain size, 3) Microbial Biomass Carbon (MB-C), 4) Chlorophyll *a* & phaeopigments). Samples for sediment density and water content were collected similarly but not split to retain the mass to volume ratio of the samples. Porewater samples were collected using Rhizon samplers (MacroRhizon, SoilMoisture Equipment Corp., CA, USA). For logistical reasons only ten Rhizon samplers were available. To avoid cross contamination these were split into designated treatment and control samplers. As an additional precaution, sampler and syringe were rinsed twice with DI water and twice with sample water before each sample was taken. Subsurface samples were taken by vertically submersing the Rhizon in the sediment until only the connector and the syringe remained. Surface samples were taken by horizontally sliding the Rhizon under the surface as shallow as possible while ensuring complete coverage of the sampler by sediment. To allow for a reasonable amount of porewater to be collected, the samplers were left in position for up to 20 minutes or until a total of about 90 mL was collected. The sample was then split into 2x 12 mL for nutrient analysis and 2x >30 mL for dissolved organic carbon (DOC) and total alkalinity (TA). The DOC samples were wrapped in aluminium foil to minimise light exposure. All samples were kept in the dark and on ice and until sampling was completed (max. 3 hours after collection). Sediment, porewater nutrient, and TA samples were frozen at -20°C while DOC samples were cooled at 4°C until analysis. Samples for microbial communities were collected as individual replicates using coring devices as described above (60 mL syringe, Ø 3 cm). Each core was separated into surface and subsurface fraction, and the fractions were immediately homogenised on a clean tray with a sterile spatula before being sampled into their respective tubes. Tubes were sealed with parafilm and stored on salt ice in the dark before being transferred to -80°C freezer after the sampling was completed.

**Analytical methods**

Sediment median grain size (GS) was measured using a Malvern Mastersizer 3000 (Malvern Panalytical Ltd., Malvern, UK) after removing organic matter with 10% hydrogen peroxide. Particles larger than 4 mm were removed with a sieve, and Calgon (Sodium Hexametaphosphate 20% solution) was added to aid dispersal of the particles^1^. The mud content of each sample was defined as the relative contribution of silt and clay particles of diameter < 63 µm^2,3^. Sediment chlorophyll *a* (chl *a*) and phaeophytin (phaeo) content were extracted in 90% buffered acetone and its fluorescence measured before and after acidification (0.1N HCl) on a Turner Designs 10-AU fluorometer (Turner Designs, San Jose, USA)^4^. Prior to pigment analysis, the sediment was freeze-dried using a Lyovapor L-200 from Büchi Flawil (Switzerland, settings: pressure 2mbar, temperature -50^o^C).

The loss on ignition (LOI) method was used to determine organic matter content (OM) from sediment samples. Between 6-8 g of sediment were dried (at 60^o^C for 24 h) and subsequently combusted following a two-step protocol of different temperature steps (300°C for 4 h, and 550^o^C for 4 h, respectively). The loss on ignition was calculated for each combustion using Eq. 1:

$LOI= \frac{pre combustion mass-post combustion mass}{pre combustion mass} * 100\%$	Eq. 1

Total sediment OM was calculated by adding the LOI of each individual step. The unstable OM fraction was defined by the LOI at 300°C and the stable OM fraction is defined as the LOI at 550°C^5^. Water content was measured as the mass lost after drying the sediment for 24 h at 60°C and was expressed as a percentage. Sediment density was calculated as the mass of the dry sample divided by its volume and expressed as g m^-3^.

Dissolved nutrients from the porewater samples, submerged incubation chambers, and water column samples were analysed on a Quickchem 8500 series 2 flow injection analyser (LACHAT instruments, Loveland, USA) following standard methods for nitrate/nitrite (NO_x_), ammonium (NH_4_^+^), and orthophosphate (PO_4_^3-^) analyses (QuikChem® Methods:31-107-04-1-A, 31-107-06-1-B, and 31-115-01-1-H, respectively). Porewater DOC was analysed using an Aurora 1030 Wet Oxidation TOC analyser (OI Analytics, College Station, USA) following a modified protocol for non-purgeable organic carbon (NPOC) to guarantee the complete oxidation of organic carbon from a complex saline sample matrix (sample volume: 1 mL; oxidiser volume: 8 mL; dissolved inorganic carbon purge: 4 min; DOC detection: 8 min). Total alkalinity was measured using the open cell titration method following SOP 3 of^6^ on a TitroLine® 6000/7000 auto titrator (SI Analytics, Xylem, Germany) using a certified reference material (A. Dickson, Scripps Institution of Oceanography, La Jolla, California).

Microbial biomass carbon (MB-C) was extracted following the direct extraction method by^7^. Briefly, 5 g of sediment was weighed separately into 50 mL centrifuge tubes and 20 mL of 0.5 M K_2_SO_4_ was added to each. To three subsamples, 0.5 mL of ethanol-free chloroform was added. Both the chloroform-exposed and the non-fumigated samples were capped and shaken for 30 min. After shaking, the suspensions were allowed to settle for 10 min and the supernatants were filtered through 0.7 µm filters (GF/F Whatman, Buckinghamshire, UK). For the sub-samples with chloroform, only the top 15 mL of the supernatant was filtered to reduce the amount chloroform in the filtrate. Filtrates from soils with and without chloroform were immediately bubbled with air for 30 min to remove any residual chloroform. The DOC in all filtrates was determined as described above. Chloroform labile C was calculated as the difference between the C extracted from the chloroform fumigated and the non-fumigated sample. No conversion factor (*k*_EC_) was used to convert chloroform labile C to microbial biomass C because the range of *k*_EC_ values (0.41–0.58) is used in the literature and it has not been tested which is best suited for the soils used here.

**DNA extraction and library preparation**

Total DNA was extracted from 0.21 [0.10-0.28] g of sediment (mean [range]), using the DNeasy® PowerSoil® Pro extraction kit (QIAGEN®, Hilden, Germany) following the manufacturer’s instructions. The extract quality was confirmed via gel electrophoresis, and the concentration was determined using nanodrop (NanoDrop 2000, Thermo Fisher Scientific, Waltham, Massachusetts, USA), and subsequently, each sample was standardised to 2.5 ng/µL. The V4-V5 of the 16S rRNA gene was amplified using the universal earth microbiome project primers 515F (5’-GTGYCAGCMGCCGCGGTAA-3’) and 926R (5’CCGYCAATTYMTTTRAGTTT-3’)^8^. One-step PCR of the target amplicon was performed using Nextera XT v2 index primers. Each sample was amplified in triplicate with each reaction containing 5 µL of total DNA in a final reaction volume of 20 µL.

Trimmed reads were processed following the DADA2 (*v1.28*)^9^ workflow with recommended parameters (except truncLen() which was set to 260 for forward and reverse reads). The quality-controlled sequence table was filtered to remove low prevalence taxa (below 1% total abundance or present in <1% of samples) and plastid DNA (chloroplasts/mitochondria).  Samples that contained <4,000 reads were removed. Taxonomy was assigned using the DADA2 naïve Bayesian classifier (assignTaxonomy) against SILVA v138^10^. Sequences were aligned with DECIPHER 2.30.0^11^ for phylogenetic tree construction (phangorn v2.11.1)^12^.

**Literature**

1	Singer, Anderson, Ledbetter, McCave, Jones, Wright*.* An assessment of analytical techniques for the size analysis of fine- grained sediments. *J. Sediment. Petrol.* **58**, 534–543 (1988)

2	Udden. Mechanical composition of clastic sediments. *Geol. Soc. Am. Bull.* **25**, 655–744 (1914)

3	Wentworth. A Scale of Grade and Class Terms for Clastic Sediments. *J. Geol.* **30**, 377–392 (1922)

4	Arar & Collins. *‘Method 445.0 - In Vitro Determination of Chlorophyll a and Pheophytin a in Marine and Freshwater Algae by Fluorescence*. (1997)

5	Santos-Andrade, Hatje, Arias-Ortiz, Patire, da Silva*.* Human disturbance drives loss of soil organic matter and changes its stability and sources in mangroves. *Environ. Res.* **202**, 111663 (2021)

6	Dickson, Sabine, & Christian. Guide to Best Practices for Ocean CO2 Measurements. *PICES Spec. Publ.* **3**, 191 (2007)

7	Gregorich, Wen, Voroney, Kachanoski*.* Calibration of a Rapid Direct Chloroform Extraction Method for Measuring Soil. *Soil Biol. Biochem.* **22**, 1009–1011 (1990)

8	Parada, Needham, & Fuhrman. Every base matters: Assessing small subunit rRNA primers for marine microbiomes with mock communities, time series and global field samples. *Environ. Microbiol.* **18**, 1403–1414 (2016)

9	Callahan, Sankaran, Fukuyama, McMurdie, Holmes*.* Bioconductor Workflow for Microbiome Data Analysis: from raw reads to community analyses. *F1000Research* **5**, 1492 (2016)

10	Quast, Pruesse, Yilmaz, Gerken, Schweer, Yarza, *et al.* The SILVA ribosomal RNA gene database project: improved data processing and web-based tools. *Nucleic Acids Res.* **41**, D590–D596 (2012)

11	Wright. Using DECIPHER v2.0 to analyze big biological sequence data in R. *R J.* **8**, 352–359 (2016)

12	Schliep. phangorn: phylogenetic analysis in R. *Bioinformatics* **27**, 592–593 (2011)

**Figure S1** **A** Map showing the plot and treatment layout and the study area on the east coast of the North Island of Aotearoa New Zealand. **B** Conceptual visualisation of the sampling strategy. Nine control plots and nine nutrient addition plots were sampled at the surface (0–2 cm) and the subsurface (5–7 cm). Slow-release complex fertiliser was applied at 200 g N m^−2^ at the start of the 4.5-month experimental period. Three sediment samples per plot were pooled into one composite sample to account for small-scale variability. Porewater was sampled using extraction samplers (Rhizon), with two replicates collected per plot and depth. 16S rRNA sequencing samples were collected in triplicate for each plot and depth.

**Figure S2** Environmental variables measured across depths and treatments. Boxplots show the median and the interquartile range (IQR, 25th to 75th percentile); whiskers extend to 1.5 × IQR.

**Table S1** Analysis of variance for the sedimentary variables between treatments and depths. Bold p-values indicate p < 0.05.

| **Term** | **Df** | **Sum Sq** | **Mean Sq** | ***F*** | ***p*** |  |
| --- | --- | --- | --- | --- | --- | --- |
| **Total organic matter** | | | | | |  |
| Treatment | 1 | 19.987 | 19.987 | 3.43 | 0.073 |  |
| Depth | 1 | 6.103 | 6.103 | 1.05 | 0.314 |  |
| Treatment × Depth | 1 | 0.336 | 0.336 | 0.06 | 0.812 |  |
| Residuals | 32 | 186.598 | 5.831 |  |  |  |
| **Salinity** | | | | | |  |
| Treatment | 1 | 4.340 | 4.340 | 2.49 | 0.124 |  |
| Depth | 1 | 5.840 | 5.840 | 3.35 | 0.076 |  |
| Treatment × Depth | 1 | 0.840 | 0.840 | 0.48 | 0.492 |  |
| Residuals | 32 | 55.722 | 1.741 |  |  |  |
| **Oxidation–reduction potential** | | | | | |  |
| Treatment | 1 | 361.0 | 361.0 | 1.31 | 0.260 |  |
| Depth | 1 | 177.8 | 177.8 | 0.65 | 0.427 |  |
| Treatment × Depth | 1 | 58.8 | 58.8 | 0.21 | 0.647 |  |
| Residuals | 32 | 8791.3 | 274.7 |  |  |  |
| **pH** | | | | | |  |
| Treatment | 1 | 0.1024 | 0.1024 | 1.26 | 0.270 |  |
| Depth | 1 | 0.0455 | 0.0455 | 0.56 | 0.460 |  |
| Treatment × Depth | 1 | 0.0187 | 0.0187 | 0.23 | 0.635 |  |
| Residuals | 32 | 2.6009 | 0.0813 |  |  |  |
| **Recalcitrant organic carbon** | | | | | |  |
| Treatment | 1 | 5.7580 | 5.7581 | 3.37 | 0.076 |  |
| Depth | 1 | 4.0580 | 4.0585 | 2.38 | 0.133 |  |
| Treatment × Depth | 1 | 0.0510 | 0.0508 | 0.03 | 0.864 |  |
| Residuals | 32 | 54.6360 | 1.7074 |  |  |  |
| **Labile organic carbon** | | | | | |  |
| Treatment | 1 | 5.2140 | 5.2142 | 4.25 | **0.048** |  |
| Depth | 1 | 4.1780 | 4.1784 | 3.40 | 0.074 |  |
| Treatment × Depth | 1 | 0.0990 | 0.0995 | 0.08 | 0.778 |  |
| Residuals | 32 | 39.2850 | 1.2277 |  |  |  |
| **Water content** | | | | | |  |
| Treatment | 1 | 149.65 | 149.65 | 4.68 | **0.038** |  |
| Depth | 1 | 33.02 | 33.02 | 1.03 | 0.317 |  |
| Treatment × Depth | 1 | 2.23 | 2.23 | 0.07 | 0.793 |  |
| Residuals | 32 | 1024.05 | 32.00 |  |  |  |
| **Microbial biomass carbon** | | | | | |  |
| Treatment | 1 | 55.9 | 55.9 | 3.29 | 0.080 |  |
| Depth | 1 | 29.9 | 29.9 | 1.76 | 0.195 |  |
| Treatment × Depth | 1 | 12.7 | 12.7 | 0.75 | 0.395 |  |
| Residuals | 30 | 510.1 | 17.0 |  |  |  |
| **log (median grain size)** | | | | | |  |
| Treatment | 1 | 0.1973 | 0.1973 | 0.43 | 0.515 |  |
| Depth | 1 | 0.7921 | 0.7921 | 1.74 | 0.196 |  |
| Treatment × Depth | 1 | 0.0003 | 0.0003 | 0.0006 | 0.980 |  |
| Residuals | 32 | 14.5533 | 0.4548 |  |  |  |

**Table S2** Analysis of variance for the porewater variables between treatments and depths. Where the interaction term was significant, Tukey HSD comparisons are given below the main comparison. Bold p-values indicate p < 0.05.

| **Term** | **Df** | **Sum Sq** | **Mean Sq** | ***F*** | ***p*** |  |
| --- | --- | --- | --- | --- | --- | --- |
| **log (NO_x_)** | | | | | |  |
| Treatment | 1 | 6.217 | 6.217 | 10.75 | **0.002** |  |
| Depth | 1 | 0.221 | 0.221 | 0.38 | 0.538 |  |
| Treatment × Depth | 1 | 0.124 | 0.124 | 0.21 | 0.645 |  |
| Residuals | 71 | 41.069 | 0.578 |  |  |  |
| **log (NH_4_^+^)** | | | | | |  |
| Treatment | 1 | 2.520 | 2.520 | 2.20 | 0.142 |  |
| Depth | 1 | 8.148 | 8.148 | 7.12 | **0.009** |  |
| Treatment × Depth | 1 | 0.389 | 0.389 | 0.34 | 0.562 |  |
| Residuals | 71 | 81.297 | 1.145 |  |  |  |
| **Dissolved organic carbon** | | | | | |  |
| Treatment | 1 | 500040 | 500040 | 2.44 | 0.128 |  |
| Depth | 1 | 257052 | 257052 | 1.26 | 0.271 |  |
| Treatment × Depth | 1 | 64860 | 64860 | 0.32 | 0.578 |  |
| Residuals | 32 | 6551980 | 204749 |  |  |  |
| **log (PO_4_^3−^)** | | | | | |  |
| Treatment | 1 | 13.607 | 13.607 | 37.14 | **< 0.001** |  |
| Depth | 1 | 13.201 | 13.201 | 36.03 | **< 0.001** |  |
| Treatment × Depth | 1 | 6.082 | 6.082 | 16.60 | **< 0.001** |  |
| Residuals | 71 | 26.011 | 0.366 |  |  |  |

**Tukey HSD, treatment × depth: log (PO_4_^3−^)**

| **Contrast** | **Difference** | **Lower CI** | **Upper CI** | ***p* adj.** |
| --- | --- | --- | --- | --- |
| Nutrient surface − control surface | 0.183 | −0.357 | 0.724 | 0.809 |
| Control subsurface − control surface | 0.284 | −0.233 | 0.800 | 0.477 |
| Nutrient subsurface − control surface | 1.611 | 1.107 | 2.115 | **< 0.001** |
| Control subsurface − nutrient surface | 0.100 | −0.440 | 0.641 | 0.961 |
| Nutrient subsurface − nutrient surface | 1.428 | 0.900 | 1.957 | **< 0.001** |
| Nutrient subsurface − control subsurface | 1.328 | 0.824 | 1.832 | **< 0.001** |

| **Term** | **Df** | **Sum Sq** | **Mean Sq** | ***F*** | ***p*** |  |  |  |  |  |
| --- | --- | --- | --- | --- | --- | --- | --- | --- | --- | --- |
| **Total alkalinity** | | | | | |  |  |  |  |  |
| Treatment | 1 | 712982 | 712982 | 4.36 | **0.045** |  |  |  |  |  |
| Depth | 1 | 2054469 | 2054469 | 12.58 | **0.001** |  |  |  |  |  |
| Treatment × Depth | 1 | 1005183 | 1005183 | 6.15 | **0.019** |  |  |  |  |  |
| Residuals | 31 | 5064021 | 163356 |  |  |  |  |  |  |  |

**Tukey HSD, treatment × depth: total alkalinity**

| **Contrast** | **Difference** | **Lower CI** | **Upper CI** | ***p* adj.** |
| --- | --- | --- | --- | --- |
| Nutrient surface − control surface | −78.4 | −611.4 | 454.7 | 0.978 |
| Control subsurface − control surface | 155.9 | −361.2 | 673.0 | 0.845 |
| Nutrient subsurface − control surface | 756.3 | 239.2 | 1273.4 | **0.002** |
| Control subsurface − nutrient surface | 234.2 | −298.8 | 767.3 | 0.636 |
| Nutrient subsurface − nutrient surface | 834.6 | 301.6 | 1367.7 | **< 0.001** |
| Nutrient subsurface − control subsurface | 600.4 | 83.3 | 1117.5 | **0.018** |

**Figure S3** Rank abundance curves of the bacterial community at each depth and treatment, with points coloured by phylum.

**Table S3** Effects of depth, treatment and their interaction on ASV richness (breakaway) and on genus-level Shannon diversity (DivNet), estimated from the non-rarefied count data. Bold p-values indicate p < 0.05.

| **Term** | **Estimate** | **Std. error** | ***p*** |  |
| --- | --- | --- | --- | --- |
| **Richness (breakaway)** | | | |  |
| Intercept | 181.45 | 10.24 | **< 0.001** |  |
| Depth (subsurface) | 99.35 | 14.71 | **< 0.001** |  |
| Treatment (nutrient) | −22.23 | 14.28 | 0.119 |  |
| Depth × Treatment | −66.65 | 20.38 | **0.001** |  |
| **Shannon diversity (DivNet)** | | | |  |
| Intercept | 3.060 | 0.006 | **< 0.001** |  |
| Depth (subsurface) | −0.170 | 0.008 | **< 0.001** |  |
| Treatment (nutrient) | −0.072 | 0.009 | **< 0.001** |  |
| Depth × Treatment | 0.150 | 0.018 | **< 0.001** |  |

**Table S4** Analysis of deviance and pairwise comparisons for the multivariate generalised linear model fitted with mvabund. Adjusted p-values are free stepdown resampling adjusted. Bold p-values indicate p < 0.05.

| **Term** | **Residual df** | **Deviance** | ***p* adj.** |
| --- | --- | --- | --- |
| Intercept | 102 | – | – |
| Treatment | 101 | 6857.5 | **0.001** |
| Depth | 100 | 8590.4 | **0.001** |
| Treatment × Depth | 99 | 4802.9 | **0.001** |

**Pairwise contrasts**

| **Contrast** | **Observed statistic** | **Adjusted *p*** |
| --- | --- | --- |
| Control surface *vs* control subsurface | 7192.6 | **0.001** |
| Nutrient surface *vs* nutrient subsurface | 5856.1 | **0.002** |
| Control subsurface *vs* nutrient subsurface | 6571.6 | **0.002** |
| Control surface *vs* nutrient surface | 5066.0 | **0.032** |

**Table S5** PERMANOVA testing the compositional differences (beta diversity) between depth and treatment. Bold p-values indicate p < 0.05.

| **Term** | **Df** | **Sum of squares** | **R²** | ***F*** | ***p*** |
| --- | --- | --- | --- | --- | --- |
| Depth × Treatment | 1 | 209.2 | 0.030 | 3.20 | **0.038** |
| Residual | 99 | 6475.0 | 0.942 | – | – |
| Total | 102 | 6870.2 | 1.000 | – | – |

**Table S6** Genera identified by differential abundance analysis (MaAsLin3, genus level, ~ Depth * Treatment + (1 | Plot)) as significantly associated with nutrient enrichment (q < 0.1) in the subsurface sediments, and genera showing a significant depth × treatment interaction, in mangrove forests of Aotearoa New Zealand. No genera showed a significant treatment effect in the surface sediments (0 of 131 genera tested, q < 0.1).

**Subsurface: treatment effect (control *vs* nutrient)**

| **Phylum** | **Order** | **Family** | **Genus** | **MaAsLin3** **coefficient** | **q-value** | **Direction** |
| --- | --- | --- | --- | --- | --- | --- |
| Proteobacteria | Chromatiales | Sedimenticolaceae | *Sedimenticola* | 1.881 | 0.005 | Nutrient |
| Desulfobacterota | Desulfobacterales | Desulfosarcinaceae | SEEP-SRB1 | −0.565 | 0.006 | Control |
| Chloroflexi | Anaerolineales | Anaerolineaceae | Unassigned.13 | −0.633 | 0.036 | Control |
| Bacteroidota | Bacteroidales | SB-5 | Unassigned.24 | −0.647 | 0.048 | Control |
| Chloroflexi | Ardenticatenales | – | Unassigned.18 | −0.760 | 0.053 | Control |
| Bacteroidota | Bacteroidetes VC2.1 Bac22 | – | Unassigned.75 | −0.512 | 0.055 | Control |
| Desulfobacterota | Desulfobacterales | Desulfosarcinaceae | Sva0081 sediment group | −0.497 | 0.072 | Control |
| Chloroflexi | SBR1031 | – | Unassigned.95 | −1.257 | 0.084 | Control |

**Depth × treatment interaction**

| **Phylum** | **Order** | **Family** | **Genus** | **MaAsLin3** **coefficient** | **q-value** | **Direction** |
| --- | --- | --- | --- | --- | --- | --- |
| Proteobacteria | Chromatiales | Sedimenticolaceae | *Sedimenticola* | 1.932 | 0.038 | Super-additive |
| Desulfobacterota | Desulfobacterales | Desulfosarcinaceae | SEEP-SRB1 | −0.335 | 0.051 | Sub-additive |
| Nitrospirota | Nitrospirales | Nitrospiraceae | *Nitrospira* | 1.552 | 0.069 | Super-additive |
| Planctomycetota | – | – | Unassigned.82 | −2.545 | 0.082 | Sub-additive |

*–, no assignment at this rank.*

**Table S7** Analysis of variance for the nearest taxon index (NTI) and the net relatedness index (NRI) of the bacterial communities between treatments and depths. Where the interaction term was significant, Tukey HSD comparisons are given below the main comparison. Bold p-values indicate p < 0.05.

| **Term** | **Df** | **Sum Sq** | **Mean Sq** | ***F*** | ***p*** |  |
| --- | --- | --- | --- | --- | --- | --- |
| **NTI** | | | | | |  |
| Treatment | 1 | 1.88 | 1.88 | 1.39 | 0.242 |  |
| Depth | 1 | 8.98 | 8.98 | 6.64 | **0.011** |  |
| Treatment × Depth | 1 | 1.09 | 1.09 | 0.81 | 0.372 |  |
| Residuals | 99 | 133.92 | 1.35 |  |  |  |
| **sqrt (NRI + 1.5)** | | | | | |  |
| Treatment | 1 | 3.610 | 3.610 | 15.54 | **< 0.001** |  |
| Depth | 1 | 6.270 | 6.270 | 26.99 | **< 0.001** |  |
| Treatment × Depth | 1 | 1.065 | 1.065 | 4.58 | **0.035** |  |
| Residuals | 99 | 22.997 | 0.232 |  |  |  |

**Tukey HSD, treatment × depth: sqrt (NRI + 1.5)**

| **Contrast** | **Difference** | **Lower CI** | **Upper CI** | ***p* adj.** |
| --- | --- | --- | --- | --- |
| Nutrient surface − control surface | −0.1823 | −0.5284 | 0.1637 | 0.517 |
| Control subsurface − control surface | 0.7032 | 0.3467 | 1.0598 | **< 0.001** |
| Nutrient subsurface − nutrient surface | 0.2962 | −0.0499 | 0.6423 | 0.121 |
| Nutrient subsurface − control subsurface | −0.5894 | −0.9459 | −0.2328 | **< 0.001** |

**Table S8** Results of the iCAMP analysis comparing bacterial community assembly mechanisms between depths and treatments. Bold p-values indicate p < 0.05.

|  | | **Depth contrast** **(subsurface *vs* surface)** | | **Treatment contrast** **(control *vs* nutrient)** | |
| --- | --- | --- | --- | --- | --- |
| **Process** | **Statistic** | **Control** | **Nutrient** | **Surface** | **Subsurface** |
| Homogenising dispersal | Relative difference | 0.091 | −0.098 | −0.062 | 0.126 |
|  | Cohen's *d* | 0.66 | −0.89 | −0.52 | 0.92 |
|  | Effect size | medium | large | medium | large |
|  | *p* | 0.316 | 0.262 | 0.350 | 0.254 |
| Dispersal limitation | Relative difference | 0.070 | −0.119 | 0.007 | 0.186 |
|  | Cohen's *d* | 1.84 | −2.85 | 0.22 | 4.97 |
|  | Effect size | large | large | small | large |
|  | *p* | 0.099 | **0.020** | 0.433 | **< 0.001** |
| Drift and others | Relative difference | −0.128 | −0.040 | −0.034 | −0.122 |
|  | Cohen's *d* | −3.84 | −1.70 | −1.55 | −3.60 |
|  | Effect size | large | large | large | large |
|  | *p* | **0.003** | 0.115 | 0.138 | **0.005** |
| Homogeneous selection | Relative difference | 0.293 | 0.354 | 0.194 | 0.119 |
|  | Cohen's *d* | 3.57 | 5.50 | 2.58 | 1.43 |
|  | Effect size | large | large | large | large |
|  | *p* | **0.001** | **< 0.001** | **0.035** | 0.156 |
| Heterogeneous selection | Relative difference | 0.265 | 0.414 | −0.195 | −0.358 |
|  | Cohen's *d* | 2.17 | 1.91 | −1.18 | −1.70 |
|  | Effect size | large | large | large | large |
|  | *p* | 0.064 | 0.078 | 0.202 | 0.111 |
| Stochasticity | Relative difference | −0.058 | −0.066 | −0.022 | −0.014 |
|  | Cohen's *d* | −3.78 | −5.96 | −2.54 | −0.82 |
|  | Effect size | large | large | large | large |
|  | *p* | **0.001** | **< 0.001** | **0.038** | 0.287 |
